# Nurses' Remuneration and Justice for All

**Published:** 1919-12-06

**Authors:** 


### THE MATRONS' AND SISTERS' DEPARTMENT.

### NURSES' REMUNERATION AND JUSTICE FOR ALL.

It has become essential that before the year 1920 has lasted three months every reputable hospital throughout the United Kingdom which advertises for probationers, and claims to train and certificate them as competent nurses, shall publish the rate of remuneration paid to every member and grade of nurses throughout its establishment. The public is properly and wisely anxious that the remuneration of nurses in every hospital which appeals to it for money shall be at least adequate, and preferably generous. About one hundred of the most reputable hospitals have already put their house in order by raising the rates of remuneration to sums which a few years ago would have been held to be impossible. These hospitals have led the van and taken the right and patriotic course, which is the course of justice and honour. No hospital worthy of the name in the United Kingdom should underpay its nurses, who, for the most part, have been, and some are, grievously and sometimes excessively overworked, some of them suffering in health, as they cannot fail to do under the conditions which prevail. Unless they are treated in a just and liberal spirit it is in the interests of the public and the sick, as well as of the nurses, that steps should be taken to close those hospitals which are in default in regard to the policy of justice for every member of the nursing staff, including the full recognition of their claims, as of those of all working women, to be employed under conditions which confine their working hours to a reasonable number and guarantee to every one of them ample relaxation with every possible facility for the employment of that relaxation under conditions "which ensure to the utmost possible extent the maximum of health" and adequate, reposeful, and health-giving hours.

We have delayed calling the attention of the hospital world to the points here raised, and impressing upon all hospital committees everywhere that their institution, wherever it be placed, whether it is under the voluntary system or under the Poor-law, must change materially and eradicate the evils of any old bad system which may have prevailed, and may even still prevail, in regard to nurses' hours, food, accommodation and salaries. The reports which appear in the newspapers up and down the country of proceedings at the Boards of Guardians and other meetings, when the claim of the nurses is advanced, are sometimes highly discreditable to all connected with Poor-law methods. The extraordinary part about this abuse, for it is an abuse, by the Guardians of the trust placed in them by the public, which has elected them to a seat on the Board, is that recently women have opposed adequate remuneration for nurses and shown their striking incompetence to fulfil the duties of a Guardian. To-day we confine ourselves to generalities, and do not publish specific instances of the breaches and serious derelictions of duty here referred to. We propose to celebrate the Year of Victory by steadily and continuously publishing all evidence which may exist of those institutions and Boards, which fail to recognise the claims of the nurse and to provide for the adequate remuneration and housing, just hours of service and relaxation, and everything which collectively makes up the full equipment of an up-to-date voluntary hospital of the very best. The publication of a list of institutions where the old evils, or any of them, continue to exist as affecting the nurses of any or all grades should speedily secure the introduction of new and adequate scales of remuneration, the reduction of the hours on and the increase of those off duty, together with an adequate supply of the best food, the proper arrangement of night-duty, sleeping accommodation, bathing facilities and other matters essential to the maintenance of the nurses' robust health. Any hospital or Poor-law establishment which professes to undertake the training and certification of nurses, and the provision of efficient nurses of the best, as attendants on the sick in the cure-houses, or, through the private staffs, in the homes of the people should be inspected and reported upon periodically. We invite the co-operation of members of the nursing profession throughout the country, and if individual nurses will join hands with the Editor in this crusade to secure prompt justice and the best of treatment for the working nurse, there is certainly good hope that good results will be attained throughout the United Kingdom by the end of March 1920. Hospital managers, and matrons, sisters, and all who are genuinely interested in the welfare of their nurses, and nurses generally, will, we hope, co-operate, for by so doing and supplying all the facts known to them we shall be enabled to bring before the public the improved conditions and actual state of development within the United Kingdom on the termination of the Victory Year.

